# Editorial: The regeneration and intervention of neurological tissue after acute and chronic injuries: from benchside to bedside

**DOI:** 10.3389/fneur.2026.1838518

**Published:** 2026-05-12

**Authors:** Xuan He, Siyu Zhou, Ye Li, Liang Wang, Jiannan Li

**Affiliations:** 1 Department of Orthopaedics, Peking University Third Hospital, Haidian, Beijing, China; 2 Department of Rehabilitation Science, The Hong Kong Polytechnic University, Hong Kong, Hong Kong SAR, China; 3 Division of Spine Surgery, Department of Orthopedics, The Third Affiliated Hospital, Southern Medical University, Academy of Orthopedics, Guangdong Province, Guangzhou, China; 4 Department of Orthopaedic Surgery, Standford University, Standford, CA, United States

**Keywords:** central nerve injury, medical translation, nerve function recovery, nerve function rehabilitation, peripheral nerve injury

The nervous system orchestrates sensory perception, motor execution, and higher cognition—a complexity that renders it uniquely vulnerable. Trauma, ischemia, compression, and neurodegeneration can disrupt neural circuits, often with devastating consequences for function and quality of life (1, 2). Despite advances in treating primary neurological injuries, patient outcomes remain critically dependent on functional recovery. Yet deficits frequently persist, underscoring a fundamental challenge: the limited capacity of the nervous system for self-repair (3).

Neural tissue tolerates injury poorly, responding with scarring, inflammation, and a microenvironment that actively inhibits axonal regeneration (4). For decades, the standard of care has remained largely unchanged. Autologous nerve transplantation—the gold standard for direct repair—is constrained by limited tissue availability and suboptimal outcomes (5). Alternative approaches often compensate for lost function rather than restoring it, highlighting a pressing unmet clinical need.

Recently, however, the landscape has shifted. Advances in materials science, stem cell biology, drug delivery, and physical therapeutics have sparked a wave of innovation. Biomaterials engineered to mimic the extracellular matrix support axonal regrowth (6). Cell-based therapies offer the potential to replace lost neurons and modulate the injury environment (7). Novel pharmaceuticals target regeneration-inhibiting pathways, while neuromodulation and brain–machine interfaces aim to restore function at the systems level (8).

This Research Topic captures the breadth of this evolving field, encompassing acute and chronic injuries to peripheral nerves, the spinal cord, and the brain. We invited leading researchers to share their findings in this forum with two primary goals: to provide a comprehensive overview of the current state of neural repair research, and to foster the cross-disciplinary dialogue necessary to propel the field forward. By bringing together diverse perspectives and approaches, we hope to lay a more robust foundation for future translational efforts—and ultimately, to accelerate the development of therapies that restore not just function, but quality of life, to patients suffering from neurological injury.

Qi et al. compared 2-year clinical outcomes of unilateral biportal endoscopic lumbar fusion (UBE-TLIF) and modified minimally invasive tubular lumbar fusion (MIS-TLIF) for single-level lumbar disc herniation. Both techniques demonstrated comparable efficacy in functional improvement (ODI), excellent–good rate (≈95%), and fusion rate (90%−93%). However, UBE-TLIF was associated with significantly less postoperative drainage and better early pain relief, suggesting reduced soft tissue trauma, albeit with longer operative time.

Zhou et al. implemented the WHO's Rehabilitation Competency Framework (RCF) in undergraduate neurorehabilitation training in China. Using a Delphi method, they developed an RCF-based curriculum defining entry-level competencies across five domains. Assessment revealed significant improvement in 13 of 15 competency indicators, including technical skills (+1.25 points) and empathy (+0.42 points), demonstrating the effectiveness of the RCF in rehabilitation education.

Liu et al. investigated whether limited vs. aggressive discectomy influences neurological recovery in lumbar disc herniation patients undergoing transforaminal endoscopic discectomy. Among 288 patients, no significant differences were observed in sensory or motor recovery between the two groups. However, limited discectomy had a significantly shorter operative time, supporting tissue-preserving approaches when adequate decompression can be achieved.

Yuan et al. characterized the clinical and genetic features of ATTRv-PN in China using two patients carrying p.Val50Met and p.Glu74Gly TTR variants. Both presented with distal paresthesia, axonal sensorimotor polyneuropathy, autonomic dysfunction, and subclinical cardiac involvement. Sural nerve biopsy showed nerve fiber loss and perivascular lymphocytic infiltration but no amyloid deposition.

Zhang Q. et al. analyzed 264 publications on acupuncture regulating neuroplasticity from 2005 to 2024. China dominated the field (73.86%). Keyword analysis identified AMPK signaling, BDNF, the brain–gut axis, and the brain–immune axis as research frontiers. The review calls for strengthened international collaboration and mechanistic research using multi-omics and neuroimaging.

Cui et al. compared OLIF-AF vs. MIS-TLIF in 57 patients with degenerative lumbar spondylolisthesis over a minimum 2-year follow-up. OLIF-AF showed shorter operative time, less blood loss, faster early recovery, and better early radiographic improvements, with comparable long-term efficacy and safety.

Huang et al. traced the evolution of subaxial cervical spine trauma classification systems from morphological description toward clinical decision-oriented functional assessment, covering the transition from early systems to SLIC and AO Spine classifications. Future directions include artificial intelligence for automated classification, diffusion tensor imaging for prognostication, and brain–computer interfaces for functional reconstruction.

Zhang Z. et al. investigated preoperative cervical spine function in 30 laminoplasty patients. Stronger preoperative cervical extension strength paradoxically predicted worse postoperative axial symptoms, potentially due to greater postoperative muscle strength decline and pain sensitization.

Zeng and Zeng examine inflammatory mediators in secondary spinal cord injury. Derived from immune cells, glia, and neurons, mediators such as cytokines, chemokines, and histamine act via TLR4, NF-κB, and MAPK signaling to induce neuronal apoptosis, disrupt the blood–spinal cord barrier, and promote glial scarring. The review discusses potential therapies including TNF-α inhibitors, COX-2 inhibitors, stem cells, and hormone therapy. Based mainly on preclinical evidence, it provides a theoretical foundation for anti-inflammatory SCI treatment, while acknowledging challenges such as mediator complexity and a lack of large-scale clinical trials.

Zhao et al. investigated the comorbidity prevalence of degenerative lumbar scoliosis (DLS) in 1,689 surgical LSS patients. The overall prevalence was 26.2%, increasing with age. Berjano type II (38.8%) and type I (35.0%) were most common. Spinal deformity significantly impacted clinical symptoms, with mutual correlation between coronal and sagittal imbalance.

On behalf of the guest editors, I extend our sincere gratitude to the editorial team and all publishing staffs for their diligent behind-the-scenes work. We thank all the scientists who submitted their work to this Research Topic—whether their manuscripts were accepted or not—for their years of dedication and deep commitment to advancing the field of neural repair. We are also deeply grateful to the reviewers for their selfless, rigorous, and impartial contributions. We believe that the findings and perspectives presented here will, much like action potentials, travel onward—guiding the field toward broader horizons, deeper insights, and more forward-looking directions.
